# Race, Ethnicity, Income Concentration and 10-Year Change in Urban Greenness in the United States

**DOI:** 10.3390/ijerph14121546

**Published:** 2017-12-10

**Authors:** Joan A. Casey, Peter James, Lara Cushing, Bill M. Jesdale, Rachel Morello-Frosch

**Affiliations:** 1 School of Public Health and the Department of Environmental Science, Policy and Management, University of California, Berkeley, CA 94720, USA; 2 Harvard Medical School and Harvard Pilgrim Health Care Institute Boston, Boston, MA 02215, USA; pjames@hsph.harvard.edu; 3 Department of Health Education, San Francisco State University, San Francisco, CA 94132, USA; lcushing@sfsu.edu; 4 Quantitative Health Sciences, University of Massachusetts Medical School, Worcester, MA 01605, USA; william.jesdale@umassmed.edu; 5 Department of Environmental Science, Policy and Management and School of Public Health, University of California, Berkeley, CA 94720, USA

**Keywords:** urban greenspace, neighborhood, ethnicity, socioeconomic factors, residence characteristics, environment

## Abstract

*Background:* Cross-sectional studies suggest urban greenness is unequally distributed by neighborhood demographics. However, the extent to which inequalities in greenness have changed over time remains unknown. *Methods:* We estimated 2001 and 2011 greenness using Moderate-resolution Imaging Spectroradiometer (MODIS) satellite-derived normalized difference vegetative index (NDVI) in 59,483 urban census tracts in the contiguous U.S. We fit spatial error models to estimate the association between baseline census tract demographic composition in 2000 and (1) 2001 greenness and (2) change in greenness between 2001 and 2011. *Results:* In models adjusted for population density, climatic factors, housing tenure, and Index of Concentration at the Extremes for income (ICE), an SD increase in percent White residents (a 30% increase) in 2000 was associated with 0.021 (95% CI: 0.018, 0.023) higher 2001 NDVI. We observed a stepwise reduction in 2001 NDVI with increased concentration of poverty. Tracts with a higher proportion of Hispanic residents in 2000 lost a small, statistically significant amount of greenness between 2001 and 2011 while tracts with higher proportions of Whites experienced a small, statistically significant increase in greenness over the same period. *Conclusions:* Census tracts with a higher proportion of racial/ethnic minorities, compared to a higher proportion of White residents, had less greenness in 2001 and lost more greenness between 2001 and 2011. Policies are needed to increase greenness, a health-promoting neighborhood asset, in disadvantaged communities.

## 1. Introduction

Urban greenness—or vegetation—provides an array of benefits that may promote health. Exposure to natural environments appears to stimulate healing and reduce stress [1,2,3,4]. Additional research in the U.S. and Europe indicate that urban greenspaces also provide opportunities for physical activity and social interaction while vegetation reduces exposure to noise [5,6], air pollution [7,8,9], and extreme heat [10,11]. Recent studies and reviews have found evidence that neighborhood greenness is associated with higher levels of physical activity, better mental health outcomes, less cardiovascular disease, increased birthweight, and decreased mortality [12,13,14,15].

Previous research also suggests that greenness in urban areas is not evenly distributed. Studies from Australia and Germany have found that the availability of greenspace is higher in urban neighborhoods with higher socioeconomic status (SES) [16,17]. In studies of several U.S. cities, racial/ethnic minorities and populations of lower SES were shown to have less neighborhood greenspace [18,19] and parks [20,21,22,23] than White and higher SES populations, although this is not universally the case [24,25]. This has led researchers to call for greater attention to social inequalities in access to greenspace and their implications for environmental justice and public health [26,27]. To our knowledge no studies to date have investigated changes in neighborhood greenness over time in relation to neighborhood demographics to assess whether inequalities in the distribution of greenness are decreasing or increasing.

Racial residential segregation and social inequality are fundamental causes of racial/ethnic disparities in neighborhood physical and social environments and are crucial to understanding the social drivers of environmental health disparities [28,29,30]. In previous work, we found that the probability of living in a neighborhood without tree canopy cover and with a majority of its land area covered in impervious surfaces was higher in more racially segregated metropolitan areas compared to less segregated metropolitan areas for all racial/ethnic groups in the United States [31]. Another study found that within 1528 U.S. cities, residents of color and lower-income lived in neighborhoods with less vegetation, on average, than their wealthier, white counterparts and these differences were greatest in racially and economically segregated cities [32]. These findings suggest that social inequality may play a role in shaping the availability and distribution of urban greenness in U.S. cities. One of the consequences of inequity and discrimination in the housing market has been the concentration of poverty in racial/ethnic minority neighborhoods [33]. At the same time, while racial residential segregation has declined somewhat in recent decades [34,35], economic polarization within cities has grown [36,37]. This suggests that class or income inequality may be an increasingly important dimension to consider in understanding recent trends in urban landscapes through time.

Herein, we investigated the extent to which baseline neighborhood racial/ethnic demographics and income segregation in 2000 were associated with changes in neighborhood greenness between 2001 and 2011 across metropolitan areas in the contiguous U.S. We use a remotely sensed measure of vegetation (the normalized difference vegetation index (NDVI)) to test the hypotheses that neighborhood racial/ethnic makeup is associated with baseline greenness and also influences the degree of change in greenness over time. We also explore the role of residential income concentration in structuring disparities in land cover using the Index of Concentration at the Extremes (ICE) [38] to measure the concentration of economic privilege and deprivation across census tracts within metropolitan areas. We hypothesized that neighborhoods with higher proportions of racial/ethnic minorities and higher concentrations of economic deprivation would be less green at baseline (i.e., have lower 2001 NDVI), and would experience a greater loss of greenness over the study period. We also hypothesized that the relationship between a neighborhood’s racial/ethnic makeup and the odds of living in a greener census tract would be differential by the level of neighborhood concentrated economic privilege (i.e., ICE). 

## 2. Materials and Methods

### 2.1. Data Sources

Data on self-reported racial/ethnic identity, neighborhood poverty rate, and housing tenure came from the 2000 decennial U.S. Census, which we downloaded from the National Historical Geographic Information System for the contiguous U.S. [39]. Because NDVI data for the analysis spanned 2001–2011, we standardized all data to 2010 census tract boundaries in order to ensure trends in greenness were based on consistent geographic boundaries [40]. Census tract boundaries are drawn to reflect similar population sizes, usually 2500 to 8000 individuals, who share similar socioeconomic characteristics when possible. They vary in size based on population density; in this study urban census tracts had a median size of 3.6 km^2^ (IQR: 1.5–13.6 km^2^). For comparability with prior work [31] and because greenness likely has different qualitative meaning in urban and rural areas, we restricted our analysis to census tracts in the contiguous U.S. (*n* = 59,647) located in metropolitan areas as defined by the U.S. Census Bureau with a 2000 population of ≥100,000 [41].

### 2.2. Normalized Difference Vegetation Index (NDVI)

NDVI is a commonly used measure of vegetative greenness based on remote sensing data and the light absorption characteristics of chlorophyll. Chlorophyll in plants absorbs visible light (0.4–0.7 μm) for use in photosynthesis, while leaves reflect near-infrared light (0.7–1.1 μm). NDVI calculates the ratio of the difference between near-infrared light and visible light to the sum of these two measures, and ranges from −1.0 to 1.0, with larger values indicating higher levels of vegetative density and photosynthetic activity [42]. For this study, we used NDVI data from the Moderate-resolution Imaging Spectroradiometer (MODIS) from the National Aeronautics and Space Administration’s Terra satellite. MODIS provides composite images every 16 days at a 250 m resolution. We considered this level of resolution adequate to summarize greenness of census tracts that generally encompass a much larger area. Neighborhood NDVI assessed at this spatial resolution has also been previously linked with reductions in mortality [15]. For our main analysis, we obtained NDVI measures from MODIS based on images from 12 July 2001 to 2011 (the first images available in July) because for most parts of the U.S., NDVI reaches its maximum and highest level of geographic variation during the height of summer. We used the zonal statistics procedure in ESRI ArcMap 10.4 (Redlands, CA, USA) to estimate the area-weighted average of the pixels in each census tract to estimate NDVI across the continental U.S. in 2001 and 2011. When the census tract area was smaller than a single NDVI pixel *(n* = 52) we used the value of the pixel that intersected the census tract centroid. We then subtracted the NDVI estimate in 2001 from the NDVI estimate in 2011 to get an estimate of the change in NDVI over this period. We also conducted a sensitivity analysis using an annual average NDVI measure constructed by averaging NDVI values from four images (1 January, 7 April, 12 July, and 30 September) for each tract and year (2001 and 2011).

### 2.3. Race/Ethnicity

We included five racial/ethnic groups in our analyses derived from 2000 U.S. census counts: (1) non-Hispanic Asians and Pacific Islanders; (2) non-Hispanic American Indians; (3) non-Hispanic Blacks; (4) Hispanics of any race; and (5) non-Hispanic Whites. For analysis, we created percentages of each race/ethnic group at the census tract level by dividing the number of individuals in each group by the total census tract population.

### 2.4. Neighborhood Socioeconomic Context

A variety of possible measures of neighborhood socioeconomic context exist, such as unemployment or poverty rates, or the proportion of adults with at least a high school education [43]. We elected to use the percent of renter-occupied housing units and ICE for income [38] as measures of area-level socioeconomic status. We selected ICE because it can simultaneously measure concentration of poverty and affluence (i.e., inequality).

We used a modified version of Massey’s [38] ICE in order to summarize concentration of poverty and affluence at the census tract level. Whereas prior health studies have used national thresholds to identify gradients of tracts with high proportions of poor and affluent households [44,45], we employed metropolitan area-level specific thresholds to account for regional variability in incomes and the cost of living. We calculated the 80th and 20th percentiles of metro area level income using 2000 Census household-level income data. We then defined ICE for each census tract as follows: *ICE_ij_* = (*A_ij_* – *P_ij_*)/*T_ij_* where *A_ij_* is the number households in census tract *i* whose income was ≥the 80th-percentile of income in metropolitan area *j* during the year 2000, *P_ij_* is the number of households in census tract *i* whose income was ≤the 20th-percentile of income in metro area *j* during the year 2000, and *T_ij_* is the total number of households in census tract *i* and metro area *j* during the year 2000. ICE ranges from −1 to 1, where −1 indicates all households in the census tract are poor and 1 indicates that all are affluent. For our analysis, we utilized quintiles of ICE, where the lowest quintile contains tracts with the highest concentrations of poverty and the highest quintile indicates tracts with the highest concentrations of affluence (Supplemental Figure S1).

### 2.5. Environmental Factors

We anticipated that regional patterns of rainfall and ecosystem characteristics would affect local greenness. We estimated cumulative rainfall at the county level in the 7 months preceding (January–July) summertime NDVI satellite imagery in 2001 and 2011 [46]. The sensitivity analysis with annual average NDVI as the outcome measure utilized annual average cumulative rainfall in 2001 and 2011. We also considered including an estimate of temperature, but neither average annual temperature nor the difference in temperature between the average warmest and coolest months provided much explanatory power in our analysis after adjusting for rainfall, and were not included in our final models.

We also characterized census tracts using the level I ecoregions described by Omernik [47]. Ecoregions represent broad regional classifications of ecosystems based on soil type, landform, major vegetation types, and climate. Ecoregions were assigned based on the location of census tracts’ centroids. Because some ecoregions contained only a handful of census tracts, we consolidated ecoregion 10 (North American Deserts) with ecoregion 12 (Southern Semi-Arid Highlands) and ecoregion 13 (Temperate Sierras) with ecoregion 11 (Mediterranean California). Thus, we used eight ecoregion classifications: 10/12, 11/13, 5 (Northern Forests), 6 (Northwestern Forested Mountains), 7 (Northwestern Forests), 8 (Eastern Temperate Forests), 9 (Great Plains), and 15 (Tropical Humid Forest) in our analysis.

From the original 59,647 census tracts located in a metro area containing ≥100,000 people, we excluded 55 census tracts with zero population overall or zero population for whom poverty status was determined, as well as those tracts without rented or owned housing units (*n* = 7; e.g., tracts containing only prisons). Lastly, census tracts for which 2001 or 2011 NDVI could not be determined (e.g., due to cloud cover) were also excluded (*n* = 102). This yielded 59,483 census tracts for analysis in both years.

### 2.6. Statistical Analysis

The main goals of the analysis were to estimate the association between 2000 census tract level demographic characteristics and (1) 2001 census tract level NDVI; and (2) change in census tract level NDVI between 2001 and 2011; and (3) evaluate the modifying effect of 2000 census tract level ICE for income on both NDVI measures.

First, we examined the univariate distributions and relationships of interest between race/ethnicity, housing tenure, ICE for income, and greenness using Spearman’s rank correlation coefficient and locally estimated scatterplot smoothing (LOESS) lines. We also examined the average racial/ethnic composition of tracts across levels of NDVI. This was done by calculating the average composition of tracts (*C_ij_*) within each 0.01 increment of NDVI as follows: *C_ij_* = $\frac{X_{ij}}{T_{j}}$, where *X_ij_* is the total number of individuals of race/ethnicity *i* living in a census tract of NDVI level *j* and *T_j_* is the total number of individuals living in a census tract of NDVI level *j.*

We then fit two sets of spatial error regression models [48] at the census tract level with NDVI as the dependent variable. Spatial error models do not assume independent and identically distributed errors, but rather allow errors distributed by a spatial autoregressive process. This type of model can account for residual spatial autocorrelation when units of observation are located proximally, and thus non-independently, in space. We defined neighbors as adjacent census tracts located in the four cardinal and four intercardinal directions from each other. In the primary analysis, we used variance-stabilized weights, but conducted a sensitivity analysis with row-standardized weights. All models were run using R Statistical Software 3.3.2 (R Foundation for Statistical Computing, Vienna, Austria) and the spdep and superlearner packages [49,50]. We considered associations statistically significant at the α = 0.05 level.

First, we conducted a cross-sectional analysis to evaluate the association between 2000 census tract level racial/ethnic composition and 2001 NDVI and second, a prospective analysis evaluating change in NDVI from 2001 to 2011. The main predictor of interest was percent of individuals of non-Hispanic American Indian, Asian, Black, and White and Hispanic race/ethnicity at the census tract level in 2000. We standardized these continuous variables, so that β coefficients were interpreted as the difference in NDVI associated with a standard deviation increase in the predictor variable. Initially, we adjusted models only for a priori identified ecological and climatic variables: Omernik ecoregion (8 ecoregions, included as a categorical variable) and precipitation (estimated at the county level in millimeters [mm]), included as a continuous variable). Second, we adjusted models for potential census tract level confounding variables also identified a priori: population density (individuals/km^2^, included as a continuous variable), percent of housing units that were renter-occupied (included as a continuous variable), and ICE (included as a categorical variable [quintiles]). We hypothesized that these factors might confound the relationship between race/ethnicity and NDVI because they are related to vegetation and because people of color on average live in more densely populated and less affluent neighborhoods and are less likely to own their own home. We evaluated nonlinearity in the association between race/ethnicity and NDVI by adding quadratic terms, however, only for change in NDVI for proportion White individuals did quadratic terms improve model fit, as measured by likelihood ratio testing. Therefore, for simplicity we ran linear models.

In a second analysis, we evaluated whether observed relationships between race/ethnicity and greenness differed by concentration of neighborhood poverty and affluence (i.e., quintiles of ICE for income). Because we were concerned about extrapolating to non-exchangeable data in stratified analyses, we used machine-learning derived propensity scores to prevent model extrapolation by limiting the stratified ICE analysis to exchangeable census tracts (i.e., those tracts with a non-zero probability of having greater than the mean percentage of White residents by ICE quintile) [50,51]. We implemented logistic regression to create propensity scores for either an NDVI ≥ 0.4 (cross-sectional models, a threshold previously used to differentiate between land use types and in health research [52,53]), or increased NDVI from 2001 to 2011 (prospective analysis, NDVI > 0). We excluded those census tracts with the highest and lowest 1% of the propensity scores, by quintile of ICE for income, from our analysis. In the cross-sectional analysis, we used logistic regression to estimate the odds of living in a green census tract, defined as NDVI ≥ 0.4, by race/ethnicity, stratified by quintiles of the ICE variable. In the prospective analysis, we estimated the odds of a census tract increasing in greenness, defined as a change in NDVI > 0. Examination of semivariograms indicated that the residuals from these models did not exhibit spatial autocorrelation, and thus we ran ordinary logistic regression models. 

## 3. Results

### 3.1. Population Characteristics

**Figure 1:**
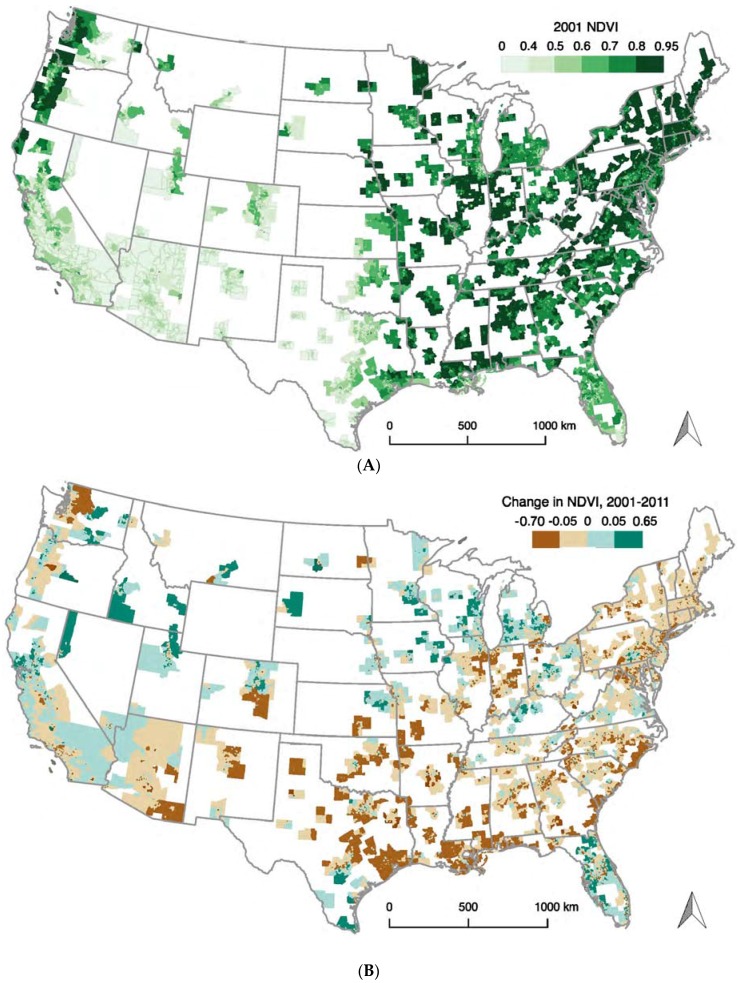
Distribution of greenness at the census tract level across the contiguous United States. (**A**) 2001 distribution of greenness (July 2001 NDVI satellite imagery); (**B**) Change in greenness (quartiles) between 2001 and 2011 (July 2001 and 2011 NDVI satellite imagery).

Our analysis included 59,483 urban U.S. census tracts, which contained 234,071,840 individuals in 2000. The average census tract in 2000 was composed of 0.9% non-Hispanic American Indians, 4.3% non-Hispanic Asians, 14.2% non-Hispanic Blacks, 66.9% non-Hispanic Whites, 13.2% Hispanics, and 0.5% other race/ethnicity (Table 1). In 2001, the average level of satellite-derived census tract greenness was 0.58 (standard deviation (SD) = 0.18) and the Northeast appeared the greenest (Figure 1A). On average, compared to 2001, census tracts became less green in 2011 (mean = −0.02 (SD = 0.06)), particularly in the southern U.S. (Table 1, Figure 1B). We observed positive correlations between the census tract proportion of White individuals and both 2001 NDVI and the change in NDVI between 2001 and 2011 (Table 1). For percent Asian, Black, and Hispanic individuals, we observed negative correlations with both 2001 and change in NDVI between 2001 and 2011. A concentration of affluence (estimated by ICE) at the census tract level was positively correlated with 2001 NDVI and change in NDVI between 2001 and 2011.

### 3.2. Bivariate Analyses

**Figure 2:**
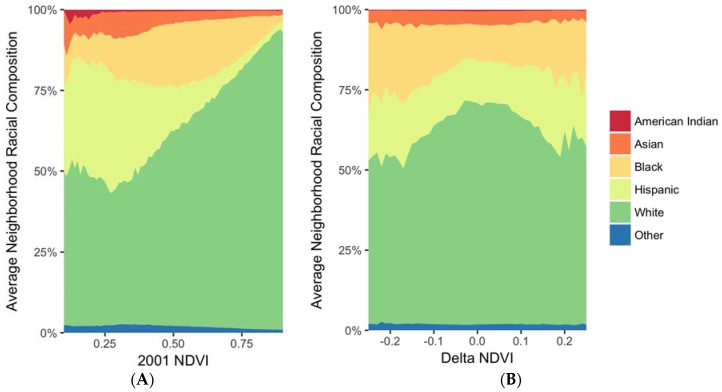
Average neighborhood composition by level of greenness in urban census tracts in the contiguous U.S. (**A**) 2000 race/ethnicity (2000 U.S. Census data) and 2001 greenness (2001 NDVI satellite imagery); (**B**) 2000 race/ethnicity (2000 U.S. Census data) and change in greenness from 2001 to 2011 (2001 and 2011 satellite imagery).

Figure 2A illustrates the relationship between average neighborhood racial compositions in the year 2000 by level of 2001 NDVI. The average census tract with NDVI = 0.58 in 2001 contained about 0.7% American Indians, 4% Asians, 17% Blacks, 66% Whites, and 11% Hispanics. As 2001 NDVI increased, the average percent of Whites also increased linearly; census tracts with NDVI = 0.90 in 2001 contained, on average, 94% Whites and 1–2% of other race/ethnicities. The relationship between 2000 racial composition and change in NDVI between 2001 and 2011 was less linear and more complex. Census tracts that lost the most greenness (i.e., change in NDVI = −0.2), were composed on average of 1% American Indians, 5% Asians, 24% Blacks, 54% Whites, and 16% Hispanics (Figure 2B). The proportion of the census tracts made up of American Indians, Asians, Hispanics and other race/ethnicities was fairly constant across changes in NDVI, while census tracts composed of relatively more Black residents had larger decreases in NDVI between 2001 and 2011 (Figure 2B).

In bivariate analyses, we found that ICE was a stronger predictor of greenness than any of the indices of neighborhood socioeconomic context that we sought as potential alternates, such as educational attainment and poverty. This provided additional rationale for including ICE as a confounding variable in our adjusted models.

### 3.3. Multivariate Analyses

In cross-sectional 2001 NDVI models, each standard deviation (SD) increase in percent American Indian (SD: 3.2%), Asian (7.4%), Black (23.5%), and Hispanic (20.0%) race/ethnicity was associated with a decrease in tract-level greenness (Table 2). Conversely, a standard deviation increase in percent White (30.2%) was associated with 0.043 (95% CI: 0.041, 0.045) higher 2001 NDVI in models only adjusted for climatic variables. The relationship between percent race/ethnicity and greenness was attenuated but still statistically significant for all race/ethnicities in models additionally adjusted for population density, ICE for income quintiles, and percent renter-occupied housing units at the census tract level. For example, a standard deviation increase in percent White was associated with 0.021 (95% CI: 0.018, 0.023) units higher 2001 NDVI in the fully adjusted model. An increase in the proportion of all other race/ethnicities was associated with lower 2001 NDVI.

In the longitudinal analyses, a standard deviation increase in 2000 percent Hispanic race/ethnicity was associated with a small but statistically significant reduction in NDVI between 2001 and 2011, while a standard deviation increase in 2000 percent White was associated with a 0.004 (95% CI: 0.002, 0.005) unit increase in NDVI in the fully adjusted model (Table 2).

In the sensitivity analysis using the annual average rather than the summertime measure of NDVI, we observed qualitatively similar results but smaller effect estimates to those shown in Table 2 (see Supplemental Table S1). One exception was that although the partially adjusted model indicated a decrease in baseline annual average 2001 NDVI per SD increase in the percentage of Black residents, this association attenuated towards the null in the fully adjusted cross-sectional model. We expected to find smaller effect estimates in the sensitivity analysis because spatial variation in greenness is most evident during the summer, when plant growth is at its peak in most of the U.S. Given the consistency in the direction of effect estimates using both measures of NDVI and the evidence that summertime NDVI was better able to capture spatial differences in greenness, we elected to use the summertime NDVI measure for the remainder of our analysis. In a second sensitivity analysis, we evaluated the influence of our choice of weights (i.e., variance-stabilized) by repeating the main analysis with row-standardized weights. We found little difference (see Supplemental Table S2).

### 3.4. Race/Ethnicity and Greenspace by Quintiles of ICE

**Figure 3:**
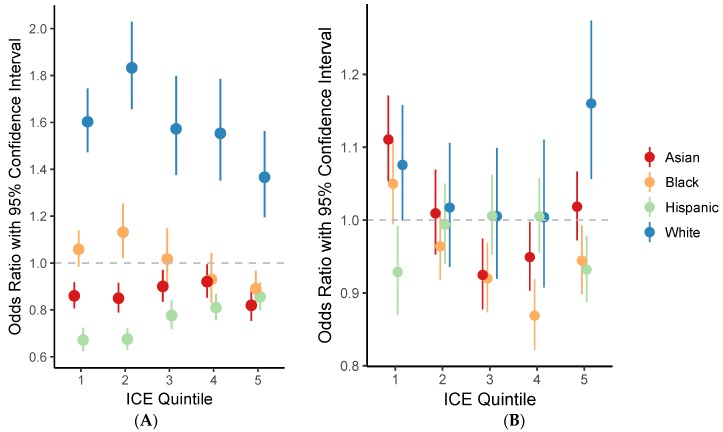
Odds ratios and 95% CIs for living in (**A**) a green census tract (2001 NDVI > 0.4) and (**B**) a census tract that became greener between 2001 and 2011 (Δ NDVI > 0) by race/ethnicity and income Index of Concentration at the Extremes (ICE) quintile (2000 U.S. Census data). The number of census tracts included in each quintile were: 1st, 10,045; 2nd, 9269; 3rd, 9229; 4th, 9211; and 5th, 9569. Models were stratified by 2000 income Index of Concentration at the Extremes (ICE) quintile, adjusted for ecoregion, precipitation (January–July 2001 for (**A**) and the difference between January–July 2011 and 2001 for (**B**)), 2000 population density, and 2000 percentage renter-occupied homes, and employed robust standard errors clustered at the county level. Native Americans were excluded from this analysis due to small numbers.

We used propensity scores derived from machine learning to restrict analysis stratified by quintile of ICE to exposed (i.e., 2001 NDVI ≥ 0.4 or change in NDVI > 0 between 2001 and 2011) and unexposed census tracts with the same probability of being exposed, based on the other variables in our models. Originally, each ICE quintile contained either 11,896 or 11,897 census tracts. After exclusions, the number of census tracts included in analysis ranged from 9211 (ICE quintile 4) to 10,045 (ICE quintile 1). The odds of being a green census tract (NDVI ≥ 0.4) consistently decreased with a 1-SD increase in percent Hispanics, but the magnitude of the odds ratio became smaller as with increasing concentration of affluence (Figure 3A). Conversely, the odds of being a green census tract consistently increased with a 1-SD increase in percent Whites, but again the magnitude of the association became smaller with increasing concentration of affluence. An SD increase in percent Blacks in the first two quintiles of ICE (i.e., concentrated poverty) was associated with increased odds of being a green census tract (1st: OR = 1.05, 95% CI: 0.99, 1.11; 2nd: OR = 1.13, 95% CI: 1.02–1.25), while an SD increase in the percent Blacks in the 5th quintile of ICE (i.e., highest concentrated affluence) was associated with significantly lower odds of being a green census tract (OR = 0.94, 95% CI: 0.89–0.99). The relationship between the percent of census tracts made up of Asian individuals and odds of being a green census tract did not appear to vary by level of ICE.

Fewer clear patterns emerged from the analysis of change in NDVI between 2001 and 2011 stratified by ICE quintile. The relationship for an SD increase in percent Whites and percent Hispanics appeared to inversely mirror one another. In the lowest and highest quintiles of ICE, an SD increase in the percent Whites was associated with increased odds of a census tract becoming greener from 2001 to 2011 and an SD increase in the percent Hispanics was associated with reduced odds of a census tract becoming greener from 2001 to 2011 (Figure 3B). There was no significant association between White or Hispanic race/ethnicity and odds of a census tract becoming greener from 2001 to 2011 in the middle 3 quintiles of ICE. For an SD increase in either percent Black or Asian residents, the odds of becoming a greener census tract from 2001 to 2011 followed a reverse-J shape across quintiles of ICE. In the lowest quintile of ICE, an SD increase in either percent Blacks or Asians was associated with increased odds of a census tract becoming greener from 2001 to 2011, however in quintiles 2–5, the odds generally declined as the percent of Black and Asian individuals increased.

## 4. Discussion

To our knowledge, this is the first study to examine the relation between cross-sectional area-level measures of racial/ethnic make-up as well as affluence and poverty concentration and both cross-sectional and temporal trends in greenness as measured by normalized difference vegetative index (NDVI). Unlike prior environmental justice analyses of disparities in urban greenness, our study sought to assess the extent to which baseline racial/ethnic and socioeconomic composition was associated with changes in greenness from 2001 to 2011, and how that relationship may have varied depending on the degree of poverty concentration within neighborhoods. Overall, in models controlling for ecological and climatic factors, population density, the proportion of renter occupied units, and the concentration of poverty or affluence, we found that tracts with a higher proportion of residents of color in 2000 tended to be less green, and in the case of Hispanics, were more likely to experience further decreases in greenness between 2001 and 2011, than tracts with higher proportions of White residents. The magnitude of these associations were moderate. For example, a SD increase in proportion White residents in 2001 was associated with about a 0.02 increase in NDVI. In the health context, a 0.1 increase in NDVI has been associated with a 20 g increase in birth weight [54] and a 12% lower rate of mortality [15]. Using annual average rather than summertime NDVI as our measure of greenness did not qualitatively change these results. Cross-sectional models stratified by Index of Concentration at the Extremes (ICE) quintiles showed that racial/ethnic disparities in 2001 NDVI persisted across levels of affluence, although differences between Hispanics compared to Whites narrowed slightly with increasing concentration of affluence. The elevated odds of increased residential greenness for White, Asian, and Black residents in communities of concentrated poverty may reflect greening of urban communities related to gentrification of poorer neighborhoods. In addition, divestments and the foreclosure crisis during the study period may have led to an increase in green space in neighborhoods of concentrated poverty, owing to an increase in the number of vacant properties and lack of development. Interestingly, concentrated affluence was associated with an increase in greenness in only neighborhoods with a higher proportion of White individuals in 2000. Conversely, affluent neighborhoods with higher proportions of Hispanic and Black individuals had reduced odds of increased greenness. This suggests an independent relationship between race/ethnicity, affluence, and greenness.

Stratified models for changes in greenness between 2001 and 2011 yielded mixed results. An increase in the percent of White residents was associated with increased odds of tracts becoming greener in the poorest and most affluent tracts only (ICE quintiles 1 and 5), while the opposite pattern was observed for Hispanics. For Blacks and Asians, increases in the proportion of either group was associated with increased odds of a tract becoming greener at the lowest ICE quintile, but this association reversed with increasing affluence. Together, these findings suggest a complicated relationship between changes in greenness over time and the spatial concentration of income that varies depending on neighborhood racial/ethnic makeup.

Our cross-sectional results showed racial and economic disparities in exposure to greenness that coincide with other analyses [18,19,20,23,32]. Saporito et al. used 2012 NDVI data to assess the distribution of greenness with a focus on differences in exposure between racial/ethnic groups (Black versus White, Hispanic versus White, people of color versus White) and economic groups (based on ordinal household poverty levels) within cities. Despite differences between our studies in terms of approach in cross-sectional analyses, we found similar racial/ethnic disparities in exposure to greenness. Our study examined a broader range of racial/ethnic groups (we included Asians), used different geographic units of analysis (we used census tracts as opposed to census block groups) and utilized a different measure of economic segregation or inequality (we used ICE versus the entropy index) [55].

Although our study elucidated racial/ethnic and income disparities in exposures to greenness through time across diverse urban areas in the United States, our analysis did not identify the fundamental drivers of these observed disparities. Moreover, NDVI imagery cannot fully capture differences in the quality of vegetation across neighborhoods, for example, distinguishing between well-maintained recreation areas and parks versus vacant lots that are overgrown with weeds. Therefore, it is possible that high amounts of greenness or temporal increases in greenness in predominantly poor neighborhoods in cities such as Detroit or Baltimore, for example, may be reflective of the increased number of vacant lots due to decades of municipal abandonment and divestment [24]. We assessed associations nationwide and did not assess regional or state-specific variability in relationships. Future research should consider heterogeneity by smaller units that may have resulted from state policy or single city greening efforts. 

Our analysis also did not include information on how much time residents spend each day within their neighborhood (i.e., census tract) and the extent to which they interacted with or used greenspace nearby. Additional analysis is needed to better distinguish the relative accessibility of greenspace in urban and suburban areas. For example, the use of greenspace by diverse populations is not only determined by its location in a neighborhood, but is also influenced by the presence of sidewalks to ensure that residents can safely walk there with young children. 

Future investigations should work to elucidate potential disparities in built and social environment attributes, such as walkability, perceptions of safety, and private versus public access rights, that affect the use patterns of greenspace across different racial/ethnic and socioeconomic groups [56,57]. Given significant demographic shifts related to changes in land use, housing construction and urban gentrification that is fueling the increased suburbanization of people of color and the poor [58], future analyses should also systematically explore relationships between simultaneous changes in neighborhood demographics and greenness within and between metropolitan regions in the United States to determine whether the suburbanization of poverty may shift sociodemographic patterns of exposure to greenness in the future.

This analysis has a number of strengths which contribute significantly to the body of literature on disparities in access to greenness. First, we analyzed data from a large sample including census tracts from all urban areas across the continental U.S. to examine whether baseline demographic factors were associated with neighborhood greenness and changes in greenness over a ten-year period. Next, our analyses were adjusted for socioeconomic, ecological, and climatic variables, as well as population density and housing tenure, which reduces the likelihood that observed associations can be explained by confounding. Finally, this analysis incorporated an innovative measure of social inequality through the use of ICE. Unlike other place-based measures of absolute levels of poverty and affluence that would provide the same coefficient if a neighborhood were 100% low-income or 100% high income, ICE provides directional tendency toward an extreme and distinguishes between concentrations of very low or very high income at the neighborhood level [59]. In addition, ICE can be applied at the census tract level, as opposed to other inequality measures that are more appropriately applied to larger geographic scales, such the Gini coefficient [60]. We also modified the ICE measure by incorporating metro-area level income thresholds to reflect regional differences in incomes and cost of living across the U.S.

## 5. Conclusions

Research has documented the public health benefits and value of parks and vegetation in metropolitan areas. However, our study builds upon a body of evidence showing that such amenities are often inequitably distributed across diverse communities in urban regions of the United States, and that these disparities are not improving. City planners and public health practitioners can better integrate urban sustainability, health, and social equity goals through investment, funding allocations, and other incentives for greenspace development that promote healthier neighborhoods and living environments in disadvantaged communities.

## Figures and Tables

**Table 1:** Summary statistics of demographics (2000) and environmental data, contiguous U.S. census tracts in metropolitan areas with population ≥100,000 (N = 59,483).

Census Tract Level Variable	Mean (SD)	Spearman’s ρ with 2001 July NDVI	Spearman’s ρ with Δ July NDVI ^a^
Population density (persons/km^2^)	2216 (4098)	−0.53 *	−0.07 *
Race/ethnicity (%)			
Non-Hispanic			
American Indian	0.9 (3.2)	−0.18 *	0.13 *
Asian	4.3 (7.4)	−0.32 *	−0.02 *
Black	14.2 (23.5)	−0.11 *	−0.12 *
White	66.9 (30.2)	0.52 *	0.09 *
Hispanic	13.2 (20.0)	−0.62 *	−0.05 *
Renter-occupied housing units (%)	32.1 (21.8)	−0.39 *	−0.05 *
Index of Concentration at the Extremes for income	0.08 (0.28)	0.27 *	0.04 *
Precipitation (mm)			
January–July 2001	2.5 (0.9)	0.52 *	−0.30 *
January–July 2011	2.8 (1.3)	0.53 *	−0.03 *
Omernik ecoregion			
Eastern Temperate Forests	0.63 (0.48)	0.52 *	−0.12 *
Northern Forests	0.01 (0.09)	0.15 *	0
Northwestern Forested Mountains	0.01 (0.10)	0.01	0.04 *
Marine West Coast Forest	0.03 (0.16)	0.03 *	0.09 *
Great Plains	0.12 (0.33)	−0.13 *	−0.01
North American Deserts ^b^	0.06 (0.23)	−0.33 *	0.01
Mediterranean California ^c^	0.12 (0.33)	−0.42 *	0.08 *
Tropical Wet Forests	0.02 (0.14)	−0.10 *	0.08 *
Greenspace (NDVI)			
July 2001	0.58 (0.18)	1.0 *	−0.21 *
July 2011	0.55 (0.18)	0.93 *	0.11 *
Average ^d^ 2001	0.45 (0.13)	0.77 *	−0.12 *
Average ^d^ 2011	0.46 (0.14)	0.82 *	0.03 *

^a^ Change in NDVI from 2001 to 2011; ^b^ Also contains Southern Semi-Arid Highlands (*n* = 43 census tracts); ^c^ Also contains Temperate Sierras (*n* = 83 census tracts); ^d^ Calculated by averaging NDVI measured on 1 January, 7 April, 12 July, and 30 September; * *p*-value < 0.001.

**Table 2:** Association between 2000 census characteristics and summertime greenness: 2001 NDVI and change in NDVI 2001–2011, contiguous U.S. census tracts.

Variable	Model 1 ^a^	Model 2 ^b^
	β (95% CI)	β (95% CI)
**2001 NDVI**		
Race/ethnicity ^c,d^		
Non-Hispanic		
American Indian	−0.003 (−0.004, −0.002)	−0.001 (−0.001, −0.002)
Asian	−0.014 (−0.015, −0.013)	−0.009 (−0.010, −0.008)
Black	−0.013 (−0.014, −0.012)	−0.003 (−0.001, −0.004)
White	0.043 (0.041, 0.045)	0.021 (0.018, 0.023)
Hispanic	−0.028 (−0.029, −0.026)	−0.013 (−0.015, −0.011)
Index of Concentration at the Extremes for income		
Quintile 1 (highest poverty concentration)		−0.021 (−0.024, −0.018)
Quintile 2		−0.009 (−0.011, −0.006)
Quintile 3		−0.002 (−0.005, 0)
Quintile 4		0 (−0.002, 0.002)
Quintile 5 (highest affluence concentration)		Reference
**Change in NDVI (2001–2011)**		
Race/ethnicity ^c,d^		
Non-Hispanic		
American Indian	0 (0, 0.001)	0 (0, 0.001)
Asian	0 (−0.001, 0.001)	0 (−0.001, 0.001)
Black	0 (−0.001, 0)	0 (−0.001, 0)
White	0.004 (0.002, 0.006)	0.004 (0.002, 0.005)
Hispanic	−0.002 (−0.003, −0.001)	−0.002 (−0.003, −0.001)
Index of Concentration at the Extremes for income		
Quintile 1 (highest poverty concentration)		0.001 (−0.001, 0.003)
Quintile 2		0.001 (0, 0.002)
Quintile 3		−0.001 (−0.002, 0.001)
Quintile 4		−0.001 (−0.002, 0)
Quintile 5 (highest affluence concentration)		Reference

^a^ Model 1, spatial error model with variance-stabilized weights, adjusted for Omernik ecoregion (Eastern Temperate Forests was the reference group) and cumulative county-level rainfall from January–July 2001 (2001 NDVI models) or difference in cumulative rainfall between January–July 2001 and January–July 2011 (change in NDVI models); ^b^ Model 2 was additionally adjusted for 2000 census tract level variables: population density (persons/km^2^), percent renter-occupied housing units, and Index of Concentration at the Extremes for income; ^c^ Race/ethnicity β coefficients were standardized; β represents the change in NDVI for a 1-SD change in the proportion of the census tract populated by the relevant racial/ethnic group; ^d^ Reference group for American Indians, Asians, Blacks, and Hispanics was non-Hispanic Whites; Hispanics were the reference group for non-Hispanic Whites.

## Supplementary Materials

The following are available online at www.mdpi.com/1660-4601/14/12/1546/s1, Figure S1: Census tract level Index of Concentration at the Extremes (ICE) for income, 2000, Table S1: Association between 2000 census characteristics and annual average greenness: 2001 NDVI and change in NDVI 2001–2011, contiguous U.S. census tracts, and Table S2: Main analysis repeated with row-standardized weights; Association between 2000 census characteristics and summertime greenness: 2001 NDVI and change in NDVI 2001–2011, contiguous U.S. census tracts.
