# Supplementary material for: Race, Ethnicity, Income Concentration and 10-Year Change in Urban Greenness in the United States
Source: Int J Environ Res Public Health. 2017 Dec 10;14(12):1546. doi: 10.3390/ijerph14121546 (PMC5750964; doi:10.3390/ijerph14121546)
Supplement: Supplementary file 1 [file ijerph-14-01546-s001.pdf]

Supplementary Materials: Race, Ethnicity, Income Concentration and 10-Year Change in Urban Greenness in the United States

Joan A. Casey [1,*], Peter James [2], Lara Cushing [3], Bill M. Jesdale [4] and Rachel Morello-Frosch [5,*]

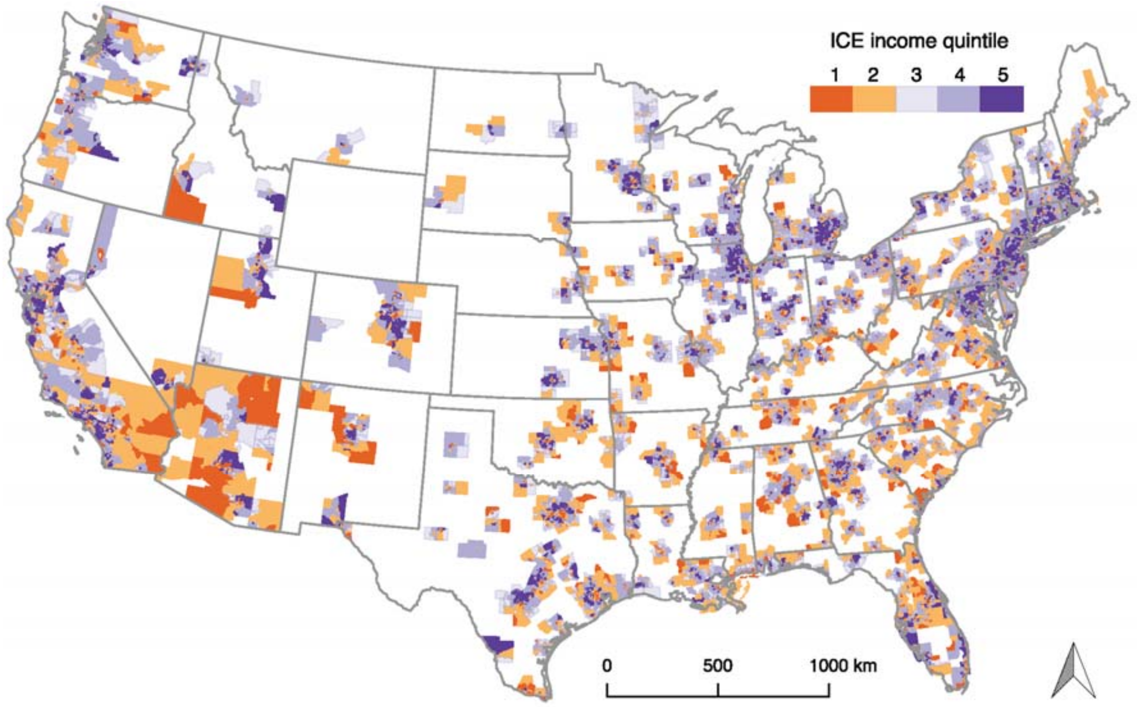

Figure S1. Census tract level Index of Concentration at the Cxtremes (ICE) for income, 2000. ICE based on metro-area 80th and 20th percentile income cut points (Quintile 1= highest poverty concentration and Quintile 5 highest affluence concentration).

Table S1. Association between 2000 census characteristics and annual average greenness: 2001 NDVI and change in NDVI 2001-2011, contiguous U.S. census tracts.

	Model 1[a]	Model 2[b]
	β (95% CI)	β (95% CI)
**2001 NDVI[c]**		
Race/ethnicity[d,e]		
Non-Hispanic		
American Indian	−0.002 (−0.003, −0.002)	0 (−0.001, 0)
Asian	−0.011 (−0.010, −0.012)	−0.007 (−0.008, −0.006)
Black	−0.008 (−0.009, −0.007)	0.001 (0, 0.002)
White	0.036 (0.034, 0.037)	0.016 (0.010, 0.014)
Hispanic	−0.023 (−0.024, −0.022)	−0.010 (−0.011, −0.009)
Index of Concentration at the Extremes for income		
Quintile 1 (highest poverty concentration)		−0.023 (−0.025, −0.020)
Quintile 2		−0.012 (−0.014, −0.010)
Quintile 3		−0.007 (−0.009, −0.005)
Quintile 4		−0.003 (0.005, −0.002)
Quintile 5 (highest affluence concentration)		Reference
**Change in NDVI (2001–2011)[c]**		
Race/ethnicity[c,d]		
Non-Hispanic		
American Indian	0 (0, 0)	0 (0, 0)
Asian	0 (0, 0)	0 (0, 0)
Black	−0.001 (−0.001, 0)	0 (−0.001, 0)
White	0.004 (0.003, 0.004)	0.002 (0.002, 0.003)
Hispanic	−0.002 (−0.003, −0.002)	−0.002 (−0.002, −0.001)
Index of Concentration at the Extremes for income		
Quintile 1 (highest poverty concentration)		−0.003 (−0.004, −0.002)
Quintile 2		−0.002 (−0.003, −0.001)
Quintile 3		−0.002 (−0.003, −0.001)
Quintile 4		−0.002 (−0.003, −0.001)
Quintile 5 (highest affluence concentration)		Reference

[a] Model 1, spatial error model with variance-stabilized weights, adjusted for Omernik ecoregion (Eastern Temperate Forests was the reference group) and annual average county-level rainfall during 2001 (2001 NDVI models) or difference in annual average county-level rainfall between 2001 and 2011 (change in NDVI models). [b] Model 2 was additionally adjusted for 2000 census tract level variables: population density (persons/km^2), percent renter-occupied housing units, and Index of Concentration at the Extremes for income. [c] Estimated from measures taken during four seasons on January 1, April 7, July 12, and September 30 in 2001 and 2011. [d] Race/ethnicity β coefficients were standardized; β represents the change in NDVI for a 1-SD change in the proportion of the census tract populated by the relevant racial/ethnic group. [e] Reference group for American Indians, Asians, Blacks, and Hispanics was non-Hispanic Whites; Hispanics were the reference group for non-Hispanic Whites

Table S2. Main analysis repeated with row-standardized weights; Association between 2000 census characteristics and summertime greenness: 2001 NDVI and change in NDVI 2001–2011, contiguous U.S. census tracts.

	Model 1[a]	Model 2[b]
	β (95% CI)	β (95% CI)
**2001 NDVI**		
Race/ethnicity[c,d]		
Non-Hispanic		
American Indian	−0.003 (−0.004, −0.002)	−0.001 (−0.001, −0.002)
Asian	−0.013 (−0.014, −0.012)	−0.008 (−0.009, −0.007)
Black	−0.013 (−0.015, −0.012)	−0.010 (−0.012, −0.009)
White	0.041 (0.039, 0.044)	0.017 (0.015, 0.019)
Hispanic	−0.027 (−0.028, −0.025)	−0.011 (−0.013, −0.001)
Index of Concentration at the Extremes for income		
Quintile 1 (highest poverty concentration)		−0.029 (−0.033, −0.026)
Quintile 2		−0.017 (−0.019, −0.014)
Quintile 3		−0.007 (−0.009, −0.005)
Quintile 4		−0.002 (0.004, 0)
Quintile 5 (highest affluence concentration)		Reference
**Change in NDVI (2001–2011)**		
Race/ethnicity[c,d]		
Non-Hispanic		
American Indian	0 (−0.003, 0.001)	0 (0, 0)
Asian	0 (−0.001, 0.001)	0 (0, 0.001)
Black	0 (−0.001, 0)	−0.001 (−0.002, 0)
White	0.003 (0.002, 0.004)	0.003 (0.002, 0.005)
Hispanic	−0.002 (−0.003, −0.001)	−0.002 (−0.003, −0.001)
Index of Concentration at the Extremes for income		
Quintile 1 (highest poverty concentration)		0.001 (−0.001, 0.003)
Quintile 2		0 (−0.002, 0.001)
Quintile 3		−0.001 (−0.002, 0)
Quintile 4		−0.001 (−0.003, 0)
Quintile 5 (highest affluence concentration)		Reference

[a] Model 1, spatial error model with row-standardized weights, adjusted for Omernik ecoregion (Eastern Temperate Forests was the reference group) and cumulative county-level rainfall from January-July 2001 (2001 NDVI models) or difference in cumulative rainfall between January-July 2001 and January-July 2011 (change in NDVI models). [b] Model 2 was additionally adjusted for 2000 census tract level variables: population density (persons/km^2), percent renter-occupied housing units, and Index of Concentration at the Extremes for income. [c] Race/ethnicity β coefficients were standardized; β represents the change in NDVI for a 1-SD change in the proportion of the census tract populated by the relevant racial/ethnic group. [d] Reference group for American Indians, Asians, Blacks, and Hispanics was non-Hispanic Whites; Hispanics were the reference group for non-Hispanic Whites

© 2017 by the authors. Licensee MDPI, Basel, Switzerland. This article is an open access article distributed under the terms and conditions of the Creative Commons Attribution (CC BY) license (http://creativecommons.org/licenses/by/4.0/).
